# Improved prognostic stratification using NCCN- and GELTAMO-international prognostic index in patients with diffuse large B-cell lymphoma

**DOI:** 10.18632/oncotarget.20988

**Published:** 2017-09-18

**Authors:** Junshik Hong, Seok Jin Kim, Myung Hee Chang, Jeong-A Kim, Jae-Yong Kwak, Jin Seok Kim, Dok Hyun Yoon, Won Sik Lee, Young Rok Do, Hye Jin Kang, Hyeon-Seok Eom, Yong Park, Jong-Ho Won, Yeung-Chul Mun, Hyo Jung Kim, Jung Hye Kwon, Jee Hyun Kong, Sung Yong Oh, Sunah Lee, Sung Hwa Bae, Deok-Hwan Yang, Hyun Jung Jun, Ho Sup Lee, Hwan Jung Yun, Soon Il Lee, Min Kyoung Kim, Jun Ho Yi, Jae Hoon Lee, Won Seog Kim, Cheolwon Suh

**Affiliations:** ^1^ Department of Internal Medicine, Seoul National University College of Medicine, Seoul, South Korea; ^2^ Department of Medicine, Samsung Medical Center, Sungkyunkwan University School of Medicine, Seoul, South Korea; ^3^ Department of Hematology-Oncology, National Health Insurance Service Ilsan Hospital, Goyang, South Korea; ^4^ Department of Internal Medicine, St. Vincent's Hospital, The Catholic University of Korea, Seoul, South Korea; ^5^ Department of Internal Medicine, Chonbuk National University Medical School & Hospital, Jeonju, South Korea; ^6^ Department of Internal Medicine, Yonsei University College of Medicine, Seoul, South Korea; ^7^ Department of Oncology, Asan Medical Center, University of Ulsan College of Medicine, Seoul, South Korea; ^8^ Department of Internal Medicine, Inje University College of Medicine, Inje University Busan Paik Hospital, Busan, South Korea; ^9^ Division of Hematology-Oncology, Department of Medicine, Dongsan Medical Center, Keimyung University School of Medicine, Daegu, South Korea; ^10^ Department of Internal Medicine, Korea Cancer Center Hospital, Korea Institute of Radiological and Medical Sciences, Seoul, South Korea; ^11^ Hematology-Oncology Clinic, National Cancer Center, Goyang, South Korea; ^12^ Department of Internal Medicine, Korea University Anam Hospital, College of Medicine, Seoul, South Korea; ^13^ Department of Internal Medicine, Soon Chun Hyang University, Seoul, South Korea; ^14^ Department of Internal Medicine, Ewha Womans University, Seoul, South Korea; ^15^ Department of Internal Medicine, Hallym University Sacred Heart Hospital, Hallym University College of Medicine, Chuncheon, South Korea; ^16^ Department of Internal Medicine, Kangdong Sacred Heart Hospital, Seoul, South Korea; ^17^ Division of Hematology-Oncology, Department of Medicine, Wonju Severance Christian Hospital, Yonsei University College of Medicine, Wonju, South Korea; ^18^ Department of Internal Medicine, Dong-A University Hospital, Busan, South Korea; ^19^ Department of Internal Medicine, Daegu Fatima Hospital, Daegu, South Korea; ^20^ Department of Internal Medicine, Catholic University of Daegu School of Medicine, Daegu, South Korea; ^21^ Department of Hemato-Oncology, Chonnam National University Hwasun Hospital, Jeollanamdo, South Korea; ^22^ Department of Internal Medicine, Seoul Medical Center, Seoul, South Korea; ^23^ Department of Internal Medicine, Kosin University Gospel Hospital, Busan, South Korea; ^24^ Department of Hemato-Oncology, Chungnam National University Hospital, Daejeon, South Korea; ^25^ Department of Internal Medicine, Dankook University College of Medicine, Cheonan, South Korea; ^26^ Department of Medicine, Yeungnam University College of Medicine, Daegu, South Korea; ^27^ Department of Internal Medicine, Chung Ang University, Seoul, South Korea; ^28^ Department of Internal Medicine, Gachon University College of Medicine, Incheon, South Korea

**Keywords:** non-hodgkin lymphoma, diffuse large B-cell lymphoma, international prognostic index, prognosis, overall survival

## Abstract

The National Comprehensive Cancer Network (NCCN)-International Prognostic Index (IPI) and GELTAMO (Grupo Español de Linfomas/Trasplante Autólogo de Médula Ósea)-IPI were developed to enable better risk prediction of patients with diffuse large B-cell lymphoma (DLBCL). The present study compared the effectiveness of risk prediction between IPI, NCCN-IPI, and GELTAMO-IPI in patients with DLBCL particularly in terms of determining high-risk patients. Among 439 patients who were enrolled to a prospective DLBCL cohort treated with R-CHOP immunochemotherapy, risk groups were classified according to the three IPIs and the prognostic significance of individual IPI factors and IPI models were analyzed and compared. All three IPI effectively separated the analyzed patients into four risk groups according to overall survival (OS). Estimated 5-year OS of patients classified as high-risk according to the IPI was 45.7%, suggesting that the IPI is limited in the selection of patients who are expected to have a poor outcome. In contrast, the 5-year OS of patients stratified as high-risk according to NCCN- and GELTAMO-IPI was 31.4% and 21.9%, respectively. The results indicate that NCCN- and GELTAMO-IPI are better than the IPI in predicting patients with poor prognosis, suggesting the superiority of enhanced, next-generation IPIs for DLBCL.

## INTRODUCTION

The International Prognostic Index (IPI) has been widely adapted in clinical practice since its introduction almost 25 years ago for patients with aggressive non-Hodgkin lymphoma (NHL) [1]. The IPI is clinically useful because it is reproducible, allows convenient scoring and categorizes patients. Several modified versions of IPI according to the subtypes of NHL have been described [2–4]. The modifications and the original IPI that comprises five factors, has been used in patients with aggressive NHL, including DLBCL [1]. The addition of rituximab to chemotherapy has improved the outcome of patients with DLBCL, and necessitated a re-evaluation of the role of the IPI. It was concluded that the IPI remains a valid prognostic indicator for patients with DLBCL in the rituximab era [5].

Despite maintaining its overall prognostic value, criticisms of the IPI are that it cannot effectively separate patients who are expected to have a poor outcome in the rituximab era [6]: contrast to pre-rituximab era, 5-year overall survival (OS) of the IPI-defined high-risk group was significantly improved, approaching 40 to 50% [7–10], suggesting that even patients classified into the poorest risk group according to the IPI have up to a 50% chance of cure. Sehn et al. reported a convergence of Kaplan-Meier curves among high-intermediate (HI) and high-risk categories defined by the IPI, and suggested a Revised-IPI (R-IPI) for better prediction of survival [7]. However, in the R-IPI, the 4-year OS of patients with high-risk category was 55%, and patients expected to have dismal prognosis were not distinguished [7].

In 2014, Zhou et al. proposed the National Comprehensive Cancer Network (NCCN)-IPI [8], which applied enhanced stratifications and scoring of age and serum lactate dehydrogenase (LDH) ratio to the upper limit of normal (ULN). In addition, they included the involvement of major extranodal organs [bone marrow, central nervous system (CNS), liver/gastrointestinal tract, and lung] as a factor of the NCCN-IPI instead of conventional definition of “involvement of >1 extranodal sites” according to the IPI. In their study using the NCCN study cohort comprising 1,650 individuals from seven NCCN centers, and the British Columbia Cancer Agency (BCCA) validation cohort (n = 1,138), the 5-year OS of NCCN-IPI-defined high-risk patients was 33% in the NCCN cohort and 38% in the BCCA cohort, suggesting the improved selection of high-risk group compared to the IPI [8]. Recently, the Grupo Español de Linfomas/Trasplante Autólogo de Médula Ósea (GELTAMO)-IPI Project Investigators proposed a new IPI incorporating the elevation of beta-2 microglobulin (B2MG) above ULN and enhanced scoring system but different from NCCN-IPI (Table 1). They reported that the GELTAMO-IPI yielded a better discrimination of high-risk DLBCL patients compared to the NCCN-IPI (5-year OS 39% vs. 49%) [9].

**Table 1:** Comparison of factors and scoring of IPI, NCCN-IPI, and GELTAMO-IPI

	IPI	Score	NCCN-IPI	Score	GELTAMO-IPI	Score
Age (years)	≤ 60	0	≤ 40	0	< 65	0
	> 60	1	41-60	1	65-79	1
			61-75	2	≥ 80	2
			> 75	3		
Ann Arbor stage	I-II	0	I-II	0	I-II	0
	III-IV	1	III-IV	1	III-IV	1
B2MG, normalized ratio	Not included		Not included		≤ 1	0
					> 1	1
ECOG performance status	0-1	0	0-1	0	0-1	0
	≥ 2	1	≥ 2	1	2	1
					3-4	2
Extranodal sites	0-1	0	No distinct sites^*^	0	Not included	
	≥ 2	1	Any distinct sites^*^	1		
Serum LDH, normalized ratio	≤ 1	0	≤ 1	0	≤ 1	0
	> 1	1	>1 to ≤ 3	1	> 1	1
			> 3	2		
Risk scoring	low	0-1	low	0-1	low	0
	Low-intermediate	2	Low-intermediate	2-3	Low-intermediate	1-3
	High-intermediate	3	High-intermediate	4-5	High-intermediate	4
	High	4-5	High	≥ 6	High	≥ 5

^*^Distinct extrnodal sites defined by NCCN-IPI: central nervous system, bone marrow, liver/gastrointestinal tract, or lung IPI, International Prognostic Index; NCCN, National Comprehensive Cancer Network; GELTAMO, Grupo Español de Linfomas/Trasplante Autólogo de Médula Ósea; B2MG, beta-2 microglobulin; ECOG, Eastern Cooperative Oncology Group; LDH, lactate dehydrogenase.

The purpose of the present study was to validate and compare the effectiveness of the risk assessment between IPI, NCCN-IPI, and GELTAMO-IPI among patients with DLBCL treated with rituximab-CHOP (R-CHOP) immunochemotherapy, particularly in terms of determining high-risk patients.

## RESULTS

### Patient characteristics and classification

**Figure 1:**
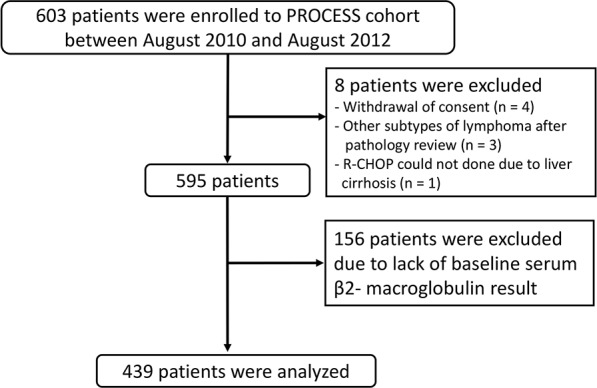
CONSORT diagram of the current study

Among 603 patients who enrolled in the PROCESS study, 164 patients were excluded [8 patients did not satisfy inclusion criteria and 156 patients lacked data of baseline serum beta-2-microglobulin (B2MG)] and the remaining 439 patients who had complete clinical, radiologic, and laboratory data enabling their classification according to the three IPI schemes were included in the current study (Figure 1).

The baseline characteristics of the analyzed patients were summarized in Table 2. Overall characteristics of the 439 patients did not deviate from those of all the patients from the PROCESS cohort. During the median follow-up duration of 55.0 months (95% CI 53.1 - 57.0), 133 patients (30.3%) underwent progression-free survival (PFS) events and 120 patients (27.3%) died. Five-year PFS and OS rates were 66.8% and 70.6%, respectively.

**Table 2:** Patient characteristics

		Analyzed patients n = 439	
		n	%
Sex	Male	250	57
Median age, years (range)		60 (20 - 89)	
Age (by NCCN-IPI)	≤ 40 years	52	12
	41- 60 years	176	40
	61-75 years	162	37
	> 75 years	49	11
Age (by GEMTAMO-IPI)	< 65 years	266	61
	65-79 years	152	35
	≥ 80 years	21	5
Performance status	ECOG 0 or 1	385	88
	ECOG 2	36	8
	ECOG 3 or 4	18	4
Ann Arbor stage	III/IV	221	50
Serum LDH	Not increased	213	49
	> ×1∼3 ULN	194	44
	> ×3 ULN	32	7
Extranodal sites (any)(No.	≥ 2 involved	158	36
Extranodal sites (by NCCN-IPI)	Involved (any)	200	46
	Bone marrow	62	14
	CNS	10	2
	Liver/GI	140	32
	Lung	30	7
Serum B2MG	Not increased	262	60
	> ×1 ULN	177	40
IPI (score)	Low (0-1)	190	43
	Low-intermediate (2)	87	20
	High-intermediate (3)	82	19
	High (4-5)	80	18
NCCN-IPI (score)	Low (0-1)	71	16
	Low-intermediate (2-3)	199	45
	High-intermediate (4-5)	130	30
	High (≥ 6)	39	9
GELTAMO-IPI (score)	Low (0)	91	21
	Low-intermediate (1-3)	269	61
	High-intermediate (4)	49	11
	High (≥5)	30	8

ECOG, Eastern Cooperative Oncology Group; LDH, Lactate dehydrogenase; IPI, International Prognostic Index; NCCN, National Comprehensive Cancer Network; B2MG, beta-2 microglobulin; GELTAMO, Grupo Español de Linfomas/Trasplante Autólogo de Médula Ósea.

**Figure 2:**
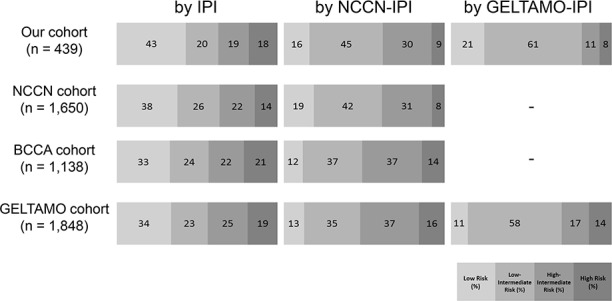
Patient distributions according to risk group in respective three IPIs

According to the IPI, the proportion of low-risk group was the highest (43%). In the NCCN-IPI, the number of low-intermediate (LI)-risk group was the highest (45%). In the GELTAMO-IPI, most patients were classified into LI-risk group (61%). Patterns of Distributio of patients according to the three IPI were overall similar with those of original NCCN and GELTAMO studies (Figure 2) [8, 9]. The NCCN- and GELTAMO-IPI classified a relatively smaller proportion of patients into the high-risk group (8.9% in NCCN-IPI and 6.8% in GELTAMO-IPI, respectively), compared to the IPI (18.2% in the high-risk group).

### Prognostic significance of individual IPI factor in the IPI, NCCN-IPI, and GELTAMO-IPI

All five factors of the IPI showed a significant difference of OS with hazard ratios (HRs) between 2.27 to 4.10 (Table 3). Enhanced stratification of age (in the NCCN- and GELTAMO-IPI), serum LDH (in the NCCN-IPI), and performance status (PS; in the GELTAMO-IPI) resulted in more effective risk stratification, except in groups between ≤ 40 *vs*. 41-60 years of age in the NCCN-IPI (*p* = 0.175). Involvement of extranodal sites designated by the NCCN-IPI failed to show prognostic significance (*p* = 0.755). Patients with an increased serum B2MG level showed significantly inferior OS compared to those with not increased B2MG. Ann Arbor staging lost its prognostic significance in the multivariate analyses performed in all three IPIs. Otherwise, most factors maintained an independent prognostic significance (Table 4).

**Table 3:** Univariate Cox regression analysis for impacts of variables from 3 IPIs on overall survival

Variables	IPI			NCCN-IPI			GELTAMO-IPI		
	HR (95% CI)	p	Score	HR (95% CI)	p	Score	HR (95% CI)	p	Score
Age (IPI)		< 0.001							
≤ 60 years	1		0						
> 60 years	3.05 (2.07 - 4.49)		1						
Age (NCCN-IPI)					< 0.001				
≤ 40 years				1		0			
41-60 years				1.96 (0.77 - 5.04)	(0.160)	1			
61-75 years				4.37 (1.75 - 10.93)	(0.002)	2			
> 75 years				9.10 (3.51 - 23.580)	(< 0.001)	3			
Age (GELTAMO-IPI)								< 0.001	
< 65 years							1		0
65-79 years							3.24 (2.20 - 4.75)	(< 0.001)	1
≥ 80 years							5.01 (2.64 - 9.49)	(< 0.001)	2
LDH (IPI- and GELTAMO-IPI)		< 0.001						< 0.001	
Not increased	1		0				1		0
Increased	2.77 (1.86 - 4.01)		1				2.77 (1.86 - 4.01)		1
LDH (NCCN-IPI)					< 0.001				
Not increased				1		0			
> 1∼3 upper limit of normal				2.20 (1.45 - 3.33)	(< 0.001)	1			
> x3 upper limit of normal				8.80 (5.19 - 14.91)	(< 0.001)	2			
ECOG PS (IPI and NCCN-IPI)		< 0.001			< 0.001				
0-1	1		0	1		0			
≥ 2	4.28 (2.86 - 6.41)		1	4.28 (2.86 - 6.41)		1			
ECOG PS (GELTAMO-IPI)								< 0.001	
0-1							1		0
2							3.16 (1.92 - 5.20)	(< 0.001)	1
3-4							8.43 (4.76 - 14.93)	(< 0.001)	2
Ann Arbor staging (common)		< 0.001			< 0.001			< 0.001	
I-II	1		0	1		0	1		0
III-IV	2.32 (1.59 - 3.40)		1	2.32 (1.59 - 3.40)		1	2.32 (1.59 - 3.40)		1
Extranodal lesion (IPI)		< 0.001							
0-1 site	1		0						
> 1 sites	2.35 (1.64 - 3.36)		1						
Extranodal sites (NCCN-IPI)					0.755				
Not involved				1		0			
Involved				1.10 (0.77 - 1.57)		1			
Serum B2MG (GELTAMO-IPI)								< 0.001	
Not increased							1		0
Increased							3.08 (2.13 - 4.47)		1

NCCN, National Comprehensive Cancer Network; GELTAMO, Grupo Español de Linfomas/Trasplante Autólogo de Médula Ósea; IPI, International Prognostic Index; LDH, lactate dehydrogenase; ECOG, Eastern Cooperative Oncology Group; B2MG, beta-2 microglobulin.

**Table 4:** Results of multivariate analysis of IPI factors in respective 3 IPIs on overall survival

	Hazard ratio (95% CI)	*p*
**IPI**		
Age > 60 years	2.75 (1.86 - 4.06)	< 0.001
LDH increased	2.03 (1.34 - 3.06)	0.001
ECOG PS ≥ 2	2.84 (1.88 - 4.30)	< 0.001
Extranodal lesion > 1 site	1.68 (1.16 - 2.44)	0.006
**NCCN-IPI**		
Age 41-60 years	1.76 (0.69 - 4.53)	0.239
Age 61-75 years	3.63 (1.45 - 9.08)	0.006
Age > 75 years	6.51 (2.48 - 17.15)	< 0.001
LDH > 1∼3 upper limit of normal	2.05 (1.34 - 3.13)	0.001
LDH > ×3 upper limit of normal	5.45 (3.11 - 9.55)	< 0.001
ECOG PS ≥ 2	2.26 (1.45 - 3.53)	< 0.001
**GELTAMO-IPI**		
Age 65-79 years	2.36 (1.57 - 3.53)	< 0.001
Age ≥ 80 years	4.07 (2.04 - 8.14)	< 0.001
LDH increased	2.08 (1.36 - 3.18)	0.001
ECOG PS 2	2.22 (1.33 - 3.69)	0.002
ECOG PS 3-4	3.93 (2.16 - 7.17)	< 0.001
Serum B2MG increased	1.55 (1.02 - 2.35)	0.039

IPI, International Prognostic Index; LDH, lactate dehydrogenase; ECOG, Eastern Cooperative Oncology Group; NCCN, National Comprehensive Cancer Network; GELTAMO, Grupo Español de Linfomas/Trasplante Autólogo de Médula Ósea; B2MG, beta-2 microglobulin.

### Stratification of patients according to the IPI, NCCN-IPI, and GELTAMO-IPI

**Figure 3:**
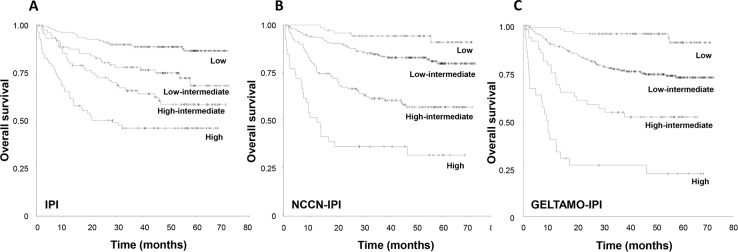
Kaplan-Meier curves for overall survival according to **(A)** IPI, **(B)** NCCN-IPI, and **(C)** GELTAMO-IPI.

All three IPI schemes effectively separated the analyzed patients into four risk groups according to OS (Table 5 and Figure 3). Estimated 5-year OS of patients classified as high-risk group according to IPI was 45.7%, suggesting that the IPI is limited in the selection of patients who are expected to have poor outcome. In contrast, the 5-year OS of patients stratified as high-risk according to NCCN- and GELTAMO-IPI were 31.4%, and 21.9%, respectively (Table 5). In the reclassification calibration statistic analysis, NCCN- and GELTAMO-IPI showed superior risk prediction (separating patients into high-risk vs. non-high-risk) compared to the IPI (Table 6). Comparison between NCCN- and GELTAMO-IPI was not statistically feasible as patient numbers of high-risk group by either of two IPIs were small and 23 patients were classified into high-risk by both NCCN- and GELTAMO-IPI.

**Table 5:** Results of risk stratification according to IPI, NCCN-IPI, and GELTAMO-IPI

*p*-value (by log-rank test)					Survival	
	L	LI	HI	H	5-year PFS	5-year OS
**IPI**						
L	-	0.002	< 0.001	< 0.001	83.9%	86.6%
LI	0.002	-	0.100	< 0.001	67.7%	68.1%
HI	< 0.001	0.100	-	0.016	56.9%	58.2%
H	< 0.001	< 0.001	0.016	-	41.1%	45.7%
**NCCN-IPI**						
L	-	0.029	< 0.001	< 0.001	89.9%	91.1%
LI	0.029	-	< 0.001	< 0.001	77.7%	79.8%
HI	< 0.001	< 0.001	-	< 0.001	54.6%	56.8%
H	< 0.001	< 0.001	< 0.001	-	26.9%	31.4%
**GELTAMO-IPI**						
L	-	< 0.001	< 0.001	< 0.001	90.8%	90.1%
LI	< 0.001	-	< 0.001	< 0.001	69.9%	72.6%
HI	< 0.001	< 0.001	-	0.001	47.9%	51.7%
H	< 0.001	< 0.001	0.001	-	19.4%	21.9%

IPI, International Prognostic Index; NCCN, National Comprehensive Cancer Network; GELTAMO, Grupo Español de Linfomas/Trasplante Autólogo de Médula Ósea.

**Table 6:** Results of reclassification calibration statistics

Comparison		χ^2^ statistics	*p*-value
IPI vs. NCCN-IPI	IPI	6.46	0.0110
	NCCN-IPI	1.69	0.1941
IPI vs. GELTAMO-IPI	IPI	10.32	0.0013
	GELTAMO-IPI	3.25	0.0712
NCCN-IPI vs. GELTAMO-IPI	NCCN-IPI	0.06	-^*^
	GELTAMO-IPI	1.60	-^*^

^*^It was impossible to calculate *p*-value because two cells have more than 20 subjects in the reclassification table.

IPI, International Prognostic Index; NCCN, National Comprehensive Cancer Network; GELTAMO, Grupo Español de Linfomas/Trasplante Autólogo de Médula Ósea.

## DISCUSSION

In the present study, the NCCN- and GELTAMO-IPI, the revised versions of the IPI that feature enhanced scoring systems (and the addition of serum B2MG in case of GELTAMO-IPI), showed improved prognostic power to detect patients with dismal prognosis compared to the IPI.

The population we analyzed reflects a real-world clinical practice of DLBCL patients because they were accrued from 27 medical centers of a nation-wide distribution, and our prospective cohort had no specific interventions relevant to patient selection or additional investigative therapy. Our patients had a median age of 60 years (57 years in the NCCN-cohort and 63 years in the BCCA-cohort of the NCCN-IPI study and 60 years in the GELTAMO-IPI study, respectively). Forty eight percent of the patients were > 60 years of age, 57% were males, 51% were LDH >1x ULN, 50% were Ann Arbor stage III or IV, and 12% of the analyzed patients were PS >1, showing that these characteristics had not significantly deviated from the populations in the original studies of the NCCN- and GELTAMO-IPI.

In the present study, involvement of the NCCN-designated extranodal sites had no prognostic significance. The prognostic implication of gastrointestinal tract, one of the designated involved sites, is controversial. Studies have suggested poor survival [11], no association [10], and even favorable outcomes [12]. In a Japanese retrospective study of 1,221 patients, the involvement of the small intestine was an IPI-independent poor prognostic factor, whereas involvement of stomach or colon was not [13]. In addition, the involvement of extranodal sites other than the NCCN-defined lesions has been suggested as a poor prognostic indicator, including the genitourinary tract [14] and female reproductive organ [10]. In the validation of the NCCN-IPI by the GELTAMO group, the NCCN-designated extranodal disease lost its prognostic value in multivariate analysis [9], and therefore it was not included in GELTAMO-IPI. A large-scale retrospective analysis (n = 25,992) using the Surveillance, Epidemiology, and End Results (SEER) database from 2004 to 2009 reported that sites of extranodal involvement are more prognostic than the number of involved sites [11]. However, a Danish-Canadian study reported that involvement of three or more extranodal sites is independently associated with dismal outcomes [10]. Considering the above results, the prognostic impact of extranodal sites in terms of its number or anatomic location is still an area of debate.

In our study, advanced Ann Arbor staging lost its prognostic significance in multivariate analyses performed in each IPI. The prognostic significance of Ann Arbor staging (I/II vs. III/IV) has been reproduced in several studies using large cohorts [8, 9, 13]. However, several lines of evidence suggest that the prognostic role of Ann Arbor staging is at least more limited than other IPI factors in the rituximab era. Ziepert et al. performed a meta-analysis of three large clinical trials [5] and reported that for patients who received rituximab-containing immunochemotherapy, Ann Arbor staging was not prognostic of OS in the MInT trial (*p* = 0.5217), MegaCHOEP study (*p* = 0.107), and RICOVER-60 trial (*p* = 0.061). Moreover, application of positron emission tomography/computed tomography (PET/CT) in response evaluation may affect the mitigation of prognostic significance of Ann Arbor staging. In the Danish-Canadian study conducted by El-Galaly et al., patients were staged and restaged by PET/CT. The authors reported no significant difference of prognosis among patients with stage I, II, and III, with only stage IV patients displaying an inferior OS. The 3-year OS were 89% [95% confidence interval (CI), 83-95%], 76% (95% CI, 62-90%), 82% (95% CI, 70-94%), and 62% (95% CI, 54-70%) for stage I, II, III, and IV disease, respectively [10]. The authors stated that the increased sensitivity of PET/CT may have upstaged a part of patients, particularly by detecting extranodal sites that would not be found by conventional CT [10]. Our study also integrated PET/CT for response evaluation. It is noteworthy that as the modality of response evaluation shift from CT to PET/CT, stage migration may occur, which may attenuate the prognostic significance of Ann Arbor staging.

Recently published studies reproduced the overall satisfactory prognostic stratification of DLBCL patients according to the NCCN-IPI in 100 to 443 DLBCL patients [10, 15–19]. However, in the aforementioned Danish-Canadian study, the NCCN-IPI was suboptimal to identify the high-risk group, showing that 3-year OS of patients with high-risk group was 48% [10]. Therefore, some modification of the NCCN-IPI, such as integrating other clinical or laboratory factors into the index, was tried to further improve the separation of patients expecting dismal outcomes. The GELTAMO-IPI was developed after a validation study of the NCCN-IPI using 2,156 patients with DLBCL from the archives of 20 hospitals in the GELTAMO network in Spain [9]. In the development of GELTAMO-IPI, enhanced scorings were used in age and PS and involvement of extranodal sites were excluded. Notably, serum B2MG was included as an IPI factor. B2MG is a component of the major histocompatibility complex class I molecule, and it is present on all nucleated cells [20]. Elevated serum B2MG has been used as a prognostic indicator in the International Staging System of multiple myeloma [21] and the Follicular Lymphoma International Prognostic Index-2 of follicular lymphoma [22], and its potential role as a prognostic biomarker was reported in many subtypes of mature lymphoid malignancies [23–26] and lymphoma-associated hemophagocytic lymphohistiocytosis [27]. The mechanism of the relationship of elevated serum B2MG to poor prognosis has been suggested, with B2MG proposed to be an indicator of heavy tumor burden with high cellular turnover rate [28]. However, this remains unclear considering that the elevation of B2MG was independent to serum LDH or Ann Arbor staging in previous studies [9, 29] as well as the present study. Further investigations are required for this issue.

In the present study, we did not integrate any biologic prognostic markers recently defined or suggested by the advance of genomics, molecular biology, or immunology in the field of DLBCL. Cell of origin [30], stromal gene signature or its protein expression [31–33], double hit [34], or co-expression of MYC and BCL2/BCL6 (double expresser) [35] were not analyzed. However, the present aim is to validate and compare IPIs, and the above integrations are beyond the scope of the study. It is limitation of our study that we could not compare the efficiency of selecting high-risk group between NCCN- and GELTAMO-IPI.

In conclusion, our study shows that NCCN- and GELTAMO-IPI have a significant advantage in predicting patients with poor prognosis, with 5-year OS rate of approximately 20 to 30%, by using basic clinical information and blood tests that are inexpensive and have a rapid turnaround time. Therefore, when selecting high-risk patients, it would be more reasonable to use NCCN- or GELTAMO-IPI rather than the IPI in clinical practice.

## MATERIALS AND METHODS

### Patients

Analyses were conducted with patients enrolled in the PROCESS (Prospective Cohort Study with Risk-Adapted Central Nervous System Evaluation in DLBCL) study from 27 hospitals belonging to the Consortium for Improving Survival of Lymphoma (CISL) in Korea. The original purpose, inclusion and exclusion criteria, and detailed information on the study conduct were as previously described [36]. Briefly, to evaluate risk factors of CNS relapse in patients with DLBCL, adult patients with newly diagnosed DLBCL planning to receive three weekly R-CHOP as a primary treatment were included. Patients with primary CNS DLBCL were excluded. Baseline evaluation of CNS involvement of DLBCL was recommended in any symptomatic patients or with features indicating a high risk for CNS involvement. However, the evaluation was not obligatory and there were no other specific interventions for the treatment of DLBCL. An interim and final response evaluation was conducted using PET/CT. The study started on August 2010 and completed patient enrollment on August 2012. Follow-up data regarding disease status and survival was updated every 6 months, with the latest update performed in February 2017. This study was approved by the institutional review boards of the participating institutions.

### IPI, NCCN-IPI, and GELTAMO IPI

Risk groups were classified according to the scores calculated as described in the IPI, NCCN-IPI, and GELTAMO IPI, respectively. For the analysis with serum LDH and B2MG levels, normalized values (ratio to the ULN of each participated institution) were calculated and used.

### Statistical analysis

PFS was time from the date of diagnosis to the date of disease progression, relapse, or last follow-up, or death from any cause. OS was defined time from diagnosis to death from any cause. Patient survival was analyzed using the Kaplan-Meier method and compared by log-rank test. Multivariate analyses by backward conditional Cox regression model were conducted with variables that had *p* < 0.1 in univariate analysis. Values were two-sided and the significance of statistics was accepted at the level of *p* < 0.05. To compare the ability to predict high-risk patients between two IPIs, the risk category of each IPI was modified into either a high-risk or a non-high-risk group (patients with low-risk + LI risk + HI risk] defined by respective IPI. A reclassification calibration statistic was used, in which an IPI with smaller statistic (χ^2^) and larger *p*-value are considered to have better risk prediction than its counterpart [37].
